# Supplementary figures and images for: Modeled buoyancy of eggs and larvae of the deep-sea shrimp Aristeus antennatus (Crustacea: Decapoda) in the northwestern Mediterranean Sea
Source: PLoS One. 2020 Jan 29;15(1):e0223396. doi: 10.1371/journal.pone.0223396 (PMC6988965; doi:10.1371/journal.pone.0223396)

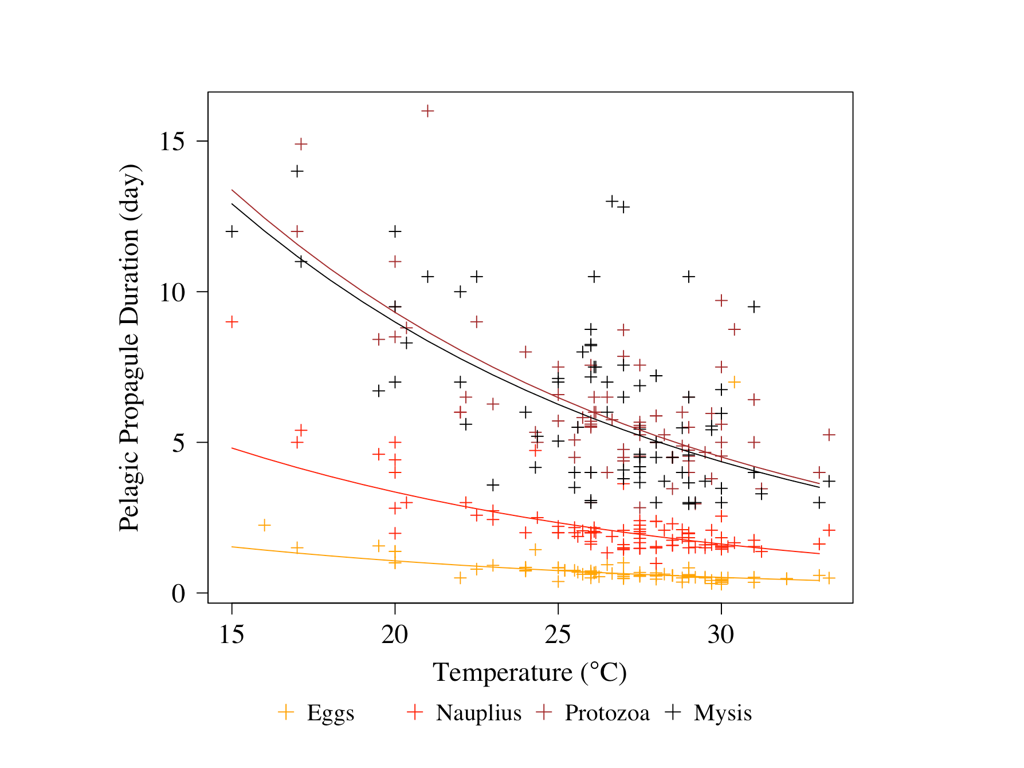

Supplement: S1 Fig — Continuous lines are the fitted exponential curves on reviewed data (cross) for each stage: eggs (orange), nauplius (red), protozoa (brown), and mysis (black). Reviewed data of PPD and temperatures were extracted from published articles and referenced in S2 File. (TIF) [file pone.0223396.s001.tif]

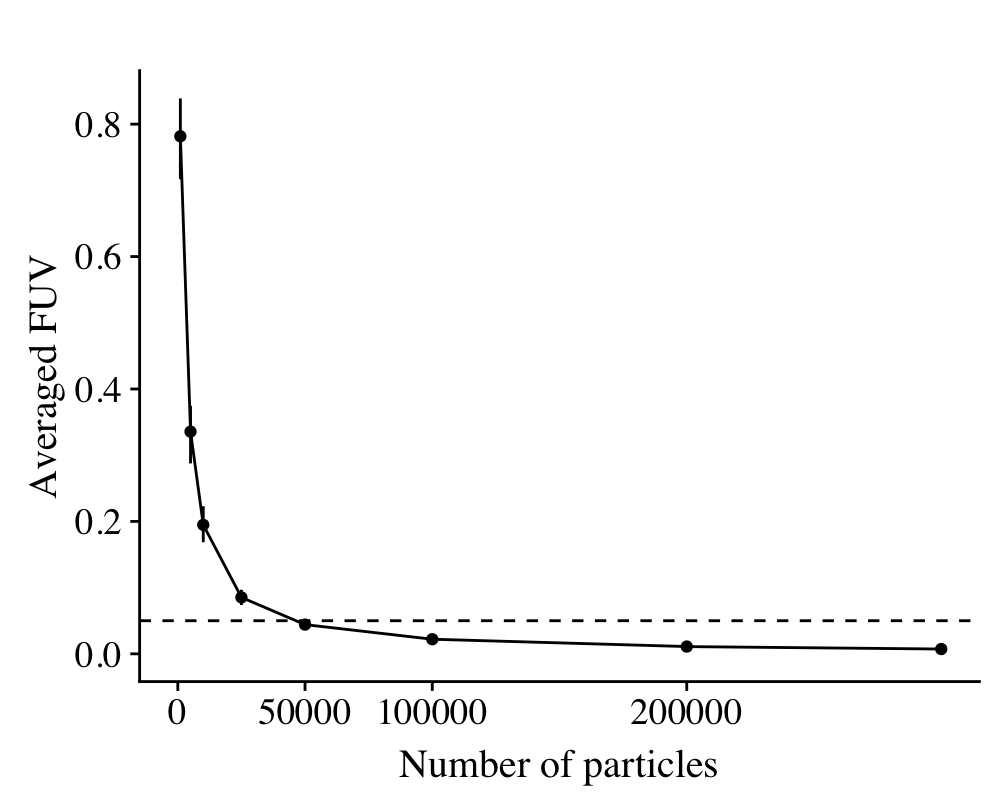

Supplement: S2 Fig — The FUV expressed the bias created by a low number of particles (FUV > 0.05) after dispersal simulation. Whiskers show the minimum and maximum of the 100 FUV calculated for each number of particles tested (1000, 2000, 5000, 10000, 50000, 100000, 200000, 300000). Horizontal dashed line is the statistical threshold of 0.05. (TIF) [file pone.0223396.s002.tif]

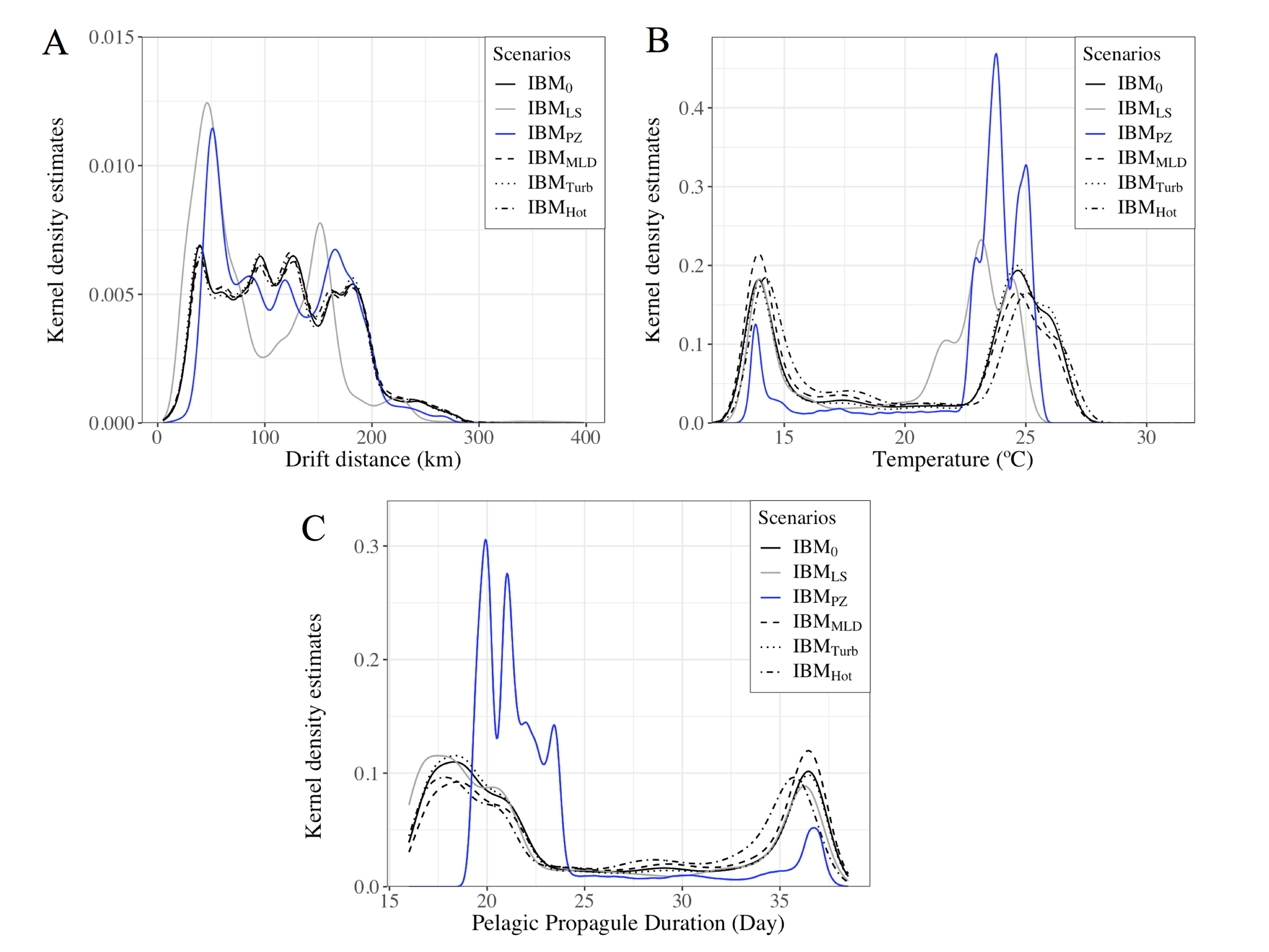

Supplement: S3 Fig — The simulated transport characteristics were A) the drift distances, B) the Pelagic Propagule Duration, and C) the water temperature during the drifts. Black lines indicate the scenarios grouped together by the PCA (Fig 3) with IBM0, IBMMLD, IBMDiff and IBMHot in four different line types. Blue line indicates the scenario IBMPZ, and grey line indicates the scenario IBMLS. See Table 1 for a description of the IBM scenarios. (TIF) [file pone.0223396.s003.tif]
