# Supplementary material for: Modeled buoyancy of eggs and larvae of the deep-sea shrimp Aristeus antennatus (Crustacea: Decapoda) in the northwestern Mediterranean Sea
Source: PLoS One. 2020 Jan 29;15(1):e0223396. doi: 10.1371/journal.pone.0223396 (PMC6988965; doi:10.1371/journal.pone.0223396)
Supplement: S1 File — Deep-sea crustaceans (Decapoda) species and taxonomically close species to Aristeus antennatus with the depth ranges of adult distribution, egg diameter and egg density. Depth range extracted from Sealife Base (https://www.sealifebase.ca) or EOL (https://eol.org). * Studied species; n/a, non available data; in grey cells, species taxonomically close to Aristeus antennatus. (DOCX) [file pone.0223396.s004.docx]

**S1 File. Information on eggs from deep-sea species and Penaeid species.** Deep-sea crustaceans (Decapoda) species and taxonomically close species to *Aristeus antennatus* with the depth ranges of adult distribution, egg diameter and egg density. Depth range extracted from Sealife Base (<https://www.sealifebase.ca>) or EOL (<https://eol.org>). * Studied species; *n/a*, non available data; in grey cells, species taxonomically close to *Aristeus antennatus*.

| **Species** | **Superfamily** | **Depth range (m)** | **Eggs diameter (µm)** | **Density (kg/m^3^)** | **Reference** |
| --- | --- | --- | --- | --- | --- |
| *Aristeus antennatus ** | Penaeoid | 173–3459 | 290–370 | n/a | [1] |
| *Aristaeomorpha foliacea* | Penaeoid | 359–461 | 359–461 | n/a | [2] |
| *Metapenaeus monoceros* | Penaeoid | 1–170 | 145–261 | n/a | [3] |
| *Metapenaeus ensis* | Penaeoid | 8–95 | 260–310 | n/a | [4] |
| Farfantep*enaeus duorarum* | Penaeoid | 0–330 | 230–320 | n/a | [5] |
| *Penaeus monodon* | Penaeoid | 0–150 | 270–280 | n/a | [6] |
| *Litopenaeus occidentalis* | Penaeoid | 2–160 | 197–238 | n/a | [7] |
| *Litopenaeus setiferus* | Penaeoid | 0–119 | 200–340 | n/a | [5] |
| *Litopenaeus stylirostris* | Penaeoid | 0–45 | 219–262 | n/a | [7] |
| Litop*enaeus vannamei* | Penaeoid | 0–72 | 271 | n/a | [8] |
| *Litopenaeus vannamei* | Penaeoid | 0–72 | 228–274 | n/a | [7] |
| *Farfantepenaeus aztecus* | Penaeoid | 0–200 | 180–280 | n/a | [5] |
| *Farfantepenaeus paulensis* | Penaeoid | 1–130 | 304 | n/a | [9] |
| *Acanthephyra acanthitelsonis* | Oplophoroid | 51.5–4434 | 640–920 | 1068 | [10] |
| *Acanthephyra acutifrons* | Oplophoroid | 357–4200 | 760–920 | 1050–1066 | [10] |
| *Acanthephyra curtirostris* | Oplophoroid | 300–5900 | 620–900 | 1057–1066 | [10] |
| *Acanthephyra pelagica* | Oplophoroid | 183–2500 | 680–1000 | 1063 | [10] |
| *Acanthephyra purpurea* | Oplophoroid | 300–3292 | 520–1120 | 1048–1075 | [10] |
| *Ephyrina bifida* | Oplophoroid | 11–5320 | 3520–4700 | 1026 | [10] |
| *Ephyrina hoskynii* | Oplophoroid | 455–2279 | 3120–4000 | 1027 | [10] |
| *Hymenodora gracilis* | Oplophoroid | 300–5400 | 2000–2300 | 1026–1030 | [10,11] |
| *Meningodora miccyla* | Oplophoroid | 400–1850 | 720–940 | 1054 | [10] |
| *Meningodora vesca* | Oplophoroid | 615–5393 | 620–1100 | 1047 | [10] |
| *Notostomus auriculatus* | Oplophoroid | 0–1200 | 640–980 | 1054 | [10] |
| *Notostomus elegans* | Oplophoroid | 450–5380 | 740–800 | 1051 | [10] |
| *Oplophorus spinosus* | Oplophoroid | 0–2700 | 3140–3210 | 1025–1031 | [10,12] |
| *Systellaspis cristata* | Oplophoroid | 100–5300 | 2720–4120 | 1028 | [10] |
| *Systellaspis braueri* | Oplophoroid | 500–2000 | 3120–4640 | 1027 | [10] |
| *Systellaspis debilis* | Oplophoroid | 0–3716 | 1880–412 | 1028–1031 | [10] |
| *Heterocarpus ensifer* | Pandalid | 57–3000 | 540–400 | 1081 | [10] |
| *Heterocarpus grimaldii* | Pandalid | 500–1473 | 520–640 | 1075 | [10] |
| *Nematocarcinus cursor* | Nematocarcinid | 542–1943 | 540–420 | 1075 | [10] |
| *Nematocarcinus exilis* | Nematocarcinid | 5230–5340 | 640–500 | 1082 | [10] |
| *Pasiphaea hoplocerca* | Pasiphaeoid | 92.5–5180 | 2240–1600 | 1041 | [10] |
| *Pasiphaea multidentata* | Pasiphaeoid | 7–4844 | 2600–2000 | 1038 | [10, 13] |
| *Plesionika edwardsii* | Pasiphaeoid | 50–850 | 440–340 | 1079 | [10] |
| *Parapasiphae sulcatifrons* | Pasiphaeoid | 500–5400 | 3200–4400 | 1026 | [10, 14] |
| *Chaceon affinis* | Geryonid | 130–2047 | 585–655 | n/a | [15] |
| *Parapontophilus talismani* | Crangonoid | n/a | 740–520 | 1076 | [10] |
| *Paralomis verrilli* | Lithodid | 850–2379 | 2000 | n/a | [16] |
| *Paromola cuvieri* | Homolid | 10–1212 | 585–655 | n/a | [17] |

**Supplementary references of S1 File**

1. Demestre M, Fortuno JM. Reproduction of the deep-water shrimp *Aristeus antennatus* (Decapoda: Dendrobranchiata). *Marine ecology progress serie*s Oldendorf. 1992; 84(1): 41–51.

2. Kapiris K, Thessalou‐legaki M. Comparative fecundity and oocyte size of *Aristaeomorpha foliacea* and *Aristeus antennatus* in the Greek Ionian Sea (E. Mediterranean) (Decapoda: Aristeidae. *Acta Zoologica.* 2006; 87(4): 239–245.

3. Nandakumar G. Reproductive biology of the speckled shrimp *Metapenaeus monoceros* (Fabricius). *Indian J. Fish*. 2001; 48(1): 1–8.

4. Ronquillo JD, Saisho T. Early developmental stages of greasyback shrimp, *Metapenaeus ensis* (de Haan, 1844) (Crustacea, Decapoda, Penaeidae*). Journal of Plankton Research*. 1993; 15(10): 1177–2106.

5. Velazquez MP, Gracia A. Fecundity of *Litopenaeus setiferus*, *Farfantepenaeus aztecu*s and *F.* *duorarum*, in the Southwestern Gulf of Mexico. *Gulf and Caribbean Research.* 2000; 12 (1): 1–9.

6. Pongtippatee-Taweepreda P, Chavadej J, Plodpai P, Pratoomchart B, Sobhon P, Weerachatyanukul W, et al. Egg activation in the black tiger shrimp *Penaeus monodon*. *Aquaculture.* 2004; 234(1–4): 183–98.

7. Rojas E, Alfaro J. In vitro manipulation of egg activation in the open thelycum shrimp *Litopenaeus. Aquaculture*. 2007; 264(1): 469–474.

8. Palacios E, Perez-Rostro CI, Ramirez JL, Ibarra AM, Racotta IS. Reproductive exhaustion in shrimp (*Penaeus vannamei*) reflected in larval biochemical composition, survival and growth. *Aquaculture*. 1999; 171(3–4): 309–321. doi: 10.1016/s0044-8486(98)00393-7

9. Peixoto S, Wasielesky W, Martino RC, Milach Â., Soares R, Cavalli RO. Comparison of reproductive output, offspring quality, ovarian histology and fatty acid composition between similarly-sized wild and domesticated. *Aquaculture.* 2008; 285(1–4): 201–206

10. Herring PJ. Observations on the embryonic development of some deep-living decapod crustaceans, with particular reference to species of Acanthephyra. *Marine Biology*. 1974; 25(1): 25–33. doi:10.1007/bf00395105

11. Chace FA. Plankton of the Bermuda Oceanographic expeditions, IX: The Bathypelagic Caridean *Crustacea. Zoologica*. 1940; 25(2): 117–209.

12. Sudnik SA. Biology of the shrimp *Oplophorus spinosus* (Brullé, 1839) (Decapoda, Oplophoridae) in the continental slope waters of the coast of northwest Africa. *Crustacean.* 2017; 90(7–10): 1235–1249

13. Apollonio S. Breeding and fecundity of the glass shrimp, *Pasiphae multidentata* (Decapoda, Caridea) in the Gulf of Maine*. J. Fish. Res. Board. Can.* 1969; 26: 1969–1983.

14. Sudnik SA, Falhenhaug T. Maturation, fecundity and embryos development in three deep-water shrimps (Decapoda: Caridea: Pasiphaeidae, Oplophoridae) along the mid-Atlantic Ridge from Iceland to the Azores. *Arthropoda Selecta.* 2015; 24(4): 401–416.

15. Tuset VM, Espinosa DI, García-Mederos A, Santana JI, González JA. Egg development and fecundity estimation in deep-sea red crab, *Chaceon affinis* (Geryonidae), off the Canary Islands (NE Atlantic*). Fisheries Research*. 2011; 109(2–3): 373–378.

16. Komai T, Amaoka K. Records of some rare deep-sea Decapod Crustaceans from the Okhotsk coast of Hokkaido (Caridea and Anomura). *Bull. Fac. Fish. Hokkaido Univ*. 1989; 40(4): 278–291.

17. Triay-Portella R, Ruiz-Díaz R, Pajuelo JG, González JA. Ovarian maturity, egg development, and offspring generation of the deep-water shrimp *Plesionika edwardsii* (Decapoda, Pandalidae) from three isolated populations in the eastern North Atlantic. *Marine Biology Research*. 2017; 13(2): 174–187.
