# Supplementary material for: Modeled buoyancy of eggs and larvae of the deep-sea shrimp Aristeus antennatus (Crustacea: Decapoda) in the northwestern Mediterranean Sea
Source: PLoS One. 2020 Jan 29;15(1):e0223396. doi: 10.1371/journal.pone.0223396 (PMC6988965; doi:10.1371/journal.pone.0223396)
Supplement: S2 File — T, water temperature (°C). (DOCX) [file pone.0223396.s005.docx]

**S2 File.** **Database on Penaeid embryonic and larval duration by stages.**  T, water temperature (ºC).

| **Species** | **T (ºC)** | **Stage duration (days)** | **Stage** | **Reference** |
| --- | --- | --- | --- | --- |
| *Artemesia longinaris* | 20.00 | 1.00 | eggs | [1] |
|  | 17.00 | 1.50 | eggs |  |
|  | 20.00 | 4.00 | Nauplius |  |
|  | 17.00 | 5.00 | Nauplius |  |
|  | 20.00 | 11.00 | Protozoa |  |
|  | 17.00 | 12.00 | Protozoa |  |
|  | 20.00 | 12.00 | Mysis |  |
|  | 17.00 | 14.00 | Mysis |  |
| *Macropetasma africanus* | 22.00 | 0.50 | eggs | [2] |
|  | 22.00 | 6.00 | Nauplius |  |
|  | 22.00 | 6.00 | Protozoa |  |
|  | 22.00 | 7.00 | Mysis |  |
| *Metapeanopsis dalei* | 25.00 | 0.84 | eggs | [3] |
|  | 25.00 | 2.00 | Nauplius |  |
|  | 25.00 | 6.58 | Protozoa |  |
|  | 25.00 | 5.04 | Mysis |  |
| *Metapeanopsis stridulans* | 25.75 | 0.62 | eggs | [4] |
|  | 25.75 | 2.06 | Nauplius |  |
|  | 25.75 | 5.82 | Protozoa |  |
|  | 25.75 | 8.00 | Mysis |  |
| *Metapenaeus affinis* | 27.50 | 0.58 | eggs | [5] |
|  | 27.50 | 2.12 | Nauplius |  |
|  | 27.50 | 5.67 | Protozoa |  |
|  | 27.50 | 6.88 | Mysis |  |
|  | 27.50 | 1.48 | Nauplius | [6] |
|  | 26.10 | 0.67 | eggs | [7] |
|  | 26.10 | 2.04 | Nauplius |  |
|  | 26.10 | 6.00 | Protozoa |  |
|  | 26.10 | 7.50 | Mysis |  |
|  | 29.70 | 0.31 | eggs | [8] |
|  | 28.80 | 0.36 | eggs |  |
|  | 29.70 | 0.42 | eggs |  |
|  | 29.70 | 1.60 | Nauplius |  |
|  | 28.80 | 1.84 | Nauplius |  |
|  | 29.70 | 2.08 | Nauplius |  |
|  | 29.70 | 3.79 | Protozoa |  |
|  | 28.80 | 4.88 | Protozoa |  |
|  | 29.70 | 5.42 | Mysis |  |
|  | 28.80 | 5.48 | Mysis |  |
|  | 29.70 | 5.54 | Mysis |  |
|  | 29.70 | 5.96 | Protozoa |  |
|  | 30.00 | 0.45 | eggs | [9] |
|  | 30.00 | 2.55 | Nauplius |  |
|  | 30.00 | 6.75 | Mysis |  |
|  | 30.00 | 7.50 | Protozoa |  |
| *Metapenaeus bennettae* | 26.00 | 0.62 | eggs | [10] |
|  | 26.00 | 2.00 | Nauplius |  |
|  | 26.00 | 5.70 | Protozoa |  |
|  | 26.00 | 8.75 | Mysis |  |
| *Metapenaeus brevicornis* | 30.00 | 0.40 | eggs | [11] |
|  | 30.00 | 1.83 | Nauplius |  |
|  | 30.00 | 5.00 | Protozoa |  |
|  | 27.00 | 0.48 | eggs | [12] |
|  | 27.00 | 1.48 | Nauplius |  |
|  | 27.00 | 8.73 | Protozoa |  |
|  | 27.00 | 12.81 | Mysis |  |
| *Metapenaeus dalli* | 26.00 | 2.00 | Nauplius | [13] |
|  | 26.00 | 4.00 | Protozoa |  |
|  | 26.00 | 4.00 | Mysis |  |
| *Metapenaeus dobsoni* | 26.15 | 0.69 | eggs | [14] |
|  | 26.15 | 2.00 | Nauplius |  |
|  | 26.15 | 6.00 | Protozoa |  |
|  | 26.15 | 7.50 | Mysis |  |
|  | 30.00 | 0.35 | eggs | [15] |
|  | 30.00 | 1.45 | Nauplius |  |
|  | 30.00 | 5.60 | Protozoa |  |
|  | 30.00 | 5.96 | Mysis |  |
| *Metapenaeus ensis* | 27.00 | 1.00 | eggs | [16] |
|  | 27.00 | 1.50 | Nauplius |  |
|  | 27.00 | 4.50 | Protozoa |  |
|  | 27.00 | 6.50 | Mysis |  |
|  | 29.00 | 0.53 | eggs | [17] |
|  | 29.00 | 1.99 | Nauplius |  |
|  | 29.00 | 3.66 | Mysis |  |
|  | 29.00 | 4.74 | Protozoa |  |
| *Metapenaeus joyneri* | 22.50 | 0.79 | eggs | [18] |
|  | 22.50 | 2.58 | Nauplius |  |
|  | 22.50 | 9.00 | Protozoa |  |
|  | 22.50 | 10.50 | Mysis |  |
|  | 22.00 | 10.00 | Mysis |  |
| *Metapenaeus macleayi* | 26.00 | 0.67 | eggs | [19] |
|  | 26.00 | 1.62 | Nauplius |  |
|  | 26.00 | 5.50 | Protozoa |  |
|  | 26.00 | 8.25 | Mysis |  |
| *Metapenaeus monoceros* | 24.00 | 0.75 | eggs | [20] |
|  | 28.00 | 0.56 | eggs |  |
|  | 32.00 | 0.47 | eggs |  |
|  | 28.50 | 1.58 | Nauplius | [21] |
|  | 27.50 | 1.48 | Nauplius | [6] |
|  | 26.10 | 0.67 | eggs | [22] |
|  | 26.10 | 2.17 | Nauplius |  |
|  | 26.10 | 6.50 | Protozoa |  |
|  | 26.10 | 10.50 | Mysis |  |
|  | 26.50 | 0.94 | eggs | [23] |
|  | 26.50 | 1.33 | Nauplius |  |
|  | 26.50 | 6.00 | Mysis |  |
|  | 26.50 | 6.50 | Protozoa |  |
| *Metapenaeus moyebi* | 26.50 | 4.00 | Protozoa | [24] |
|  | 26.50 | 7.00 | Mysis |  |
|  | 31.00 | 0.35 | eggs | [25] |
|  | 29.00 | 0.83 | eggs |  |
|  | 31.00 | 1.54 | Nauplius |  |
|  | 29.00 | 1.83 | Nauplius |  |
|  | 31.00 | 5.00 | Protozoa |  |
|  | 29.00 | 5.50 | Protozoa |  |
|  | 31.00 | 9.50 | Mysis |  |
|  | 29.00 | 10.50 | Mysis |  |
| *Parapenaeopsis stylifera* | 25.00 | 0.38 | eggs | [26] |
|  | 25.00 | 2.21 | Nauplius |  |
|  | 25.00 | 5.71 | Protozoa |  |
|  | 25.00 | 7.12 | Mysis |  |
|  | 26.65 | 0.65 | eggs | [27] |
|  | 26.65 | 1.88 | Nauplius |  |
|  | 26.65 | 5.75 | Protozoa |  |
|  | 26.65 | 13.00 | Mysis |  |
|  | 30.00 | 0.29 | eggs | [28] |
|  | 30.00 | 1.54 | Nauplius |  |
|  | 30.00 | 9.71 | Protozoa |  |
|  | 30.40 | 7.00 | eggs |  |
|  | 30.40 | 1.67 | Nauplius |  |
|  | 30.40 | 8.75 | Protozoa |  |
| *Parapenaeus longirostris* | 16.00 | 2.25 | eggs | [29] |
| *Penaeus aztecus* | 30.20 | 0.50 | eggs | [30] |
|  | 30.20 | 1.54 | Nauplius |  |
|  | 27.50 | 2.25 | Nauplius | [31] |
|  | 27.00 | 3.63 | Nauplius |  |
|  | 27.50 | 5.44 | Mysis |  |
|  | 27.50 | 7.56 | Protozoa |  |
|  | 27.00 | 7.56 | Mysis |  |
|  | 27.00 | 7.85 | Protozoa |  |
| *Penaeus brevirostris* | 27.00 | 0.54 | eggs | [32] |
|  | 27.00 | 1.44 | Nauplius |  |
|  | 27.00 | 4.08 | Mysis |  |
|  | 27.00 | 4.38 | Protozoa |  |
| *Penaeus californiensis* | 29.00 | 0.61 | eggs | [33] |
|  | 29.00 | 1.71 | Nauplius |  |
|  | 29.00 | 4.54 | Protozoa |  |
|  | 29.00 | 4.58 | Mysis |  |
| *Penaeus chinensis* | 24.30 | 1.44 | eggs | [34] |
|  | 19.50 | 1.56 | eggs |  |
|  | 24.30 | 4.17 | Mysis |  |
|  | 19.50 | 4.60 | Nauplius |  |
|  | 24.30 | 4.73 | Nauplius |  |
|  | 24.30 | 5.33 | Protozoa |  |
|  | 19.50 | 6.71 | Mysis |  |
|  | 19.50 | 8.42 | Protozoa |  |
| *Penaeus duorarum* | 28.00 | 0.58 | eggs | [35] |
|  | 26.00 | 7.17 | Mysis | [36] |
|  | 26.00 | 7.56 | Protozoa |  |
|  | 21.00 | 10.50 | Mysis |  |
|  | 21.00 | 16.00 | Protozoa |  |
|  | 30.00 | 0.50 | eggs | [37] |
|  | 30.00 | 1.56 | Nauplius |  |
|  | 30.00 | 3.47 | Mysis |  |
|  | 30.00 | 4.54 | Protozoa |  |
|  | 27.50 | 1.81 | Nauplius | [31] |
|  | 23.00 | 2.73 | Nauplius |  |
|  | 23.00 | 3.58 | Mysis |  |
|  | 27.50 | 3.67 | Mysis |  |
|  | 27.50 | 5.56 | Protozoa |  |
|  | 23.00 | 6.27 | Protozoa |  |
| *Penaeus esculentus* | 26.25 | 0.54 | eggs | [38] |
| *Penaeus indicus* | 25.60 | 0.69 | eggs | [39] |
|  | 25.60 | 1.88 | Nauplius |  |
|  | 25.60 | 5.50 | Protozoa |  |
|  | 25.60 | 5.50 | Mysis |  |
| *Penaeus japonicus* | 28.00 | 0.58 | eggs | [40] |
|  | 28.00 | 1.54 | Nauplius |  |
|  | 28.00 | 5.00 | Protozoa |  |
|  | 28.00 | 5.00 | Mysis |  |
|  | 28.00 | 1.50 | Nauplius | [41] |
|  | 28.00 | 3.00 | Mysis |  |
|  | 28.00 | 5.00 | Protozoa |  |
| *Penaeus kerathurus* | 25.20 | 0.75 | eggs | [29] |
|  | 24.00 | 0.85 | eggs |  |
|  | 20.00 | 1.38 | eggs |  |
|  | 20.00 | 2.81 | Nauplius |  |
|  | 27.50 | 2.04 | Nauplius | [42] |
|  | 27.50 | 4.00 | Mysis |  |
|  | 27.50 | 5.50 | Protozoa |  |
|  | 27.00 | 2.08 | Nauplius | [43] |
|  | 20.00 | 4.42 | Nauplius |  |
|  | 29.50 | 0.50 | eggs | [44] |
|  | 29.50 | 1.50 | Nauplius |  |
|  | 29.50 | 3.71 | Mysis |  |
|  | 29.50 | 4.67 | Protozoa |  |
|  | 24.00 | 2.00 | Nauplius | [45] |
|  | 24.00 | 6.00 | Mysis |  |
|  | 24.00 | 8.00 | Protozoa |  |
|  | 25.20 | 0.75 | eggs | [29] |
|  | 24.00 | 0.85 | eggs |  |
|  | 20.00 | 1.38 | eggs |  |
| *Penaeus latisulcatus* | 24.36 | 2.50 | Nauplius | [46] |
|  | 20.35 | 3.00 | Nauplius |  |
|  | 22.17 | 3.00 | Nauplius |  |
|  | 24.36 | 5.00 | Protozoa |  |
|  | 24.36 | 5.20 | Mysis |  |
|  | 17.12 | 5.40 | Nauplius |  |
|  | 17.12 | 11.00 | Mysis |  |
|  | 17.12 | 14.90 | Protozoa |  |
|  | 22.17 | 5.60 | Mysis |  |
|  | 22.17 | 6.50 | Protozoa |  |
|  | 20.35 | 8.30 | Mysis |  |
|  | 20.35 | 8.80 | Protozoa |  |
|  | 29.20 | 0.52 | eggs | [47] |
|  | 29.20 | 1.52 | Nauplius |  |
|  | 29.20 | 2.96 | Protozoa |  |
|  | 29.20 | 3.00 | Mysis |  |
| *Penaeus marginatus* | 25.00 | 0.83 | eggs | [48] |
| *Penaeus merguiensis* | 27.50 | 0.55 | eggs | [49] |
|  | 27.50 | 1.67 | Nauplius |  |
|  | 27.50 | 2.83 | Protozoa |  |
|  | 27.50 | 4.19 | Mysis |  |
|  | 29.00 | 4.00 | Protozoa | [50] |
|  | 29.00 | 3.00 | Mysis |  |
|  | 33.00 | 3.00 | Mysis |  |
|  | 33.00 | 4.00 | Protozoa |  |
|  | 27.00 | 0.48 | eggs | [12] |
| *Penaeus monodon* | 28.00 | 0.46 | eggs | [51] |
|  | 28.00 | 1.50 | Nauplius |  |
|  | 28.00 | 4.50 | Mysis |  |
|  | 28.00 | 5.00 | Protozoa |  |
|  | 33.00 | 0.58 | eggs | [52] |
|  | 28.00 | 0.67 | eggs |  |
|  | 23.00 | 0.92 | eggs |  |
|  | 28.00 | 0.98 | Nauplius |  |
|  | 33.00 | 1.63 | Nauplius |  |
|  | 23.00 | 2.43 | Nauplius |  |
|  | 27.50 | 1.98 | Nauplius | [53] |
|  | 27.50 | 4.50 | Mysis |  |
|  | 27.50 | 5.25 | Protozoa |  |
|  | 27.50 | 0.68 | eggs |  |
|  | 28.80 | 0.50 | eggs | [54] |
|  | 28.80 | 2.10 | Nauplius |  |
|  | 28.80 | 4.00 | Mysis |  |
|  | 28.80 | 6.00 | Protozoa |  |
| *Penaeus occidentalis* | 27.00 | 0.60 | eggs | [55] |
|  | 27.00 | 1.60 | Nauplius |  |
|  | 27.00 | 3.79 | Mysis |  |
|  | 27.00 | 4.76 | Protozoa |  |
| *Penaeus paulensis* | 30.00 | 1.50 | Nauplius | [56] |
|  | 25.00 | 2.00 | Nauplius |  |
|  | 30.00 | 3.00 | Mysis |  |
|  | 20.00 | 5.00 | Nauplius |  |
|  | 25.00 | 7.00 | Mysis |  |
|  | 25.00 | 7.5 | Protozoa |  |
|  | 15.00 | 9.00 | Nauplius |  |
|  | 20.00 | 9.50 | Protozoa |  |
|  | 20.00 | 9.50 | Mysis |  |
|  | 15.00 | 12.00 | Mysis |  |
|  | 26.00 | 0.50 | eggs | [57] |
|  | 26.00 | 2.00 | Nauplius |  |
|  | 26.00 | 3.00 | Protozoa |  |
|  | 26.00 | 3.00 | Mysis |  |
| *Penaeus penicillatus* | 31.23 | 1.38 | Nauplius | [58] |
|  | 31.23 | 3.29 | Mysis |  |
|  | 31.23 | 3.46 | Protozoa |  |
|  | 25.50 | 2.00 | Nauplius | [59] |
|  | 25.50 | 3.50 | Mysis |  |
|  | 25.50 | 4.50 | Protozoa |  |
| *Penaeus plebejus* | 26.00 | 0.73 | eggs | [19] |
|  | 26.00 | 2.00 | Nauplius |  |
|  | 26.00 | 5.54 | Protozoa |  |
|  | 26.00 | 8.21 | Mysis |  |
| *Penaeus schmidti* | 28.50 | 2.29 | Nauplius | [60] |
|  | 28.50 | 3.46 | Protozoa |  |
|  | 28.50 | 4.50 | Mysis |  |
| *Penaeus semisulcatus* | 24.00 | 0.73 | eggs | [61] |
|  | 28.00 | 0.60 | eggs |  |
|  | 32.00 | 0.48 | eggs |  |
|  | 31.00 | 0.52 | eggs | [62] |
|  | 31.00 | 1.75 | Nauplius |  |
|  | 31.00 | 4.00 | Mysis |  |
|  | 31.00 | 6.42 | Protozoa |  |
|  | 28.00 | 0.58 | eggs | [63] |
|  | 28.00 | 2.38 | Nauplius |  |
|  | 28.00 | 5.88 | Protozoa |  |
|  | 28.00 | 7.21 | Mysis |  |
|  | 25.50 | 0.75 | eggs | [64] |
|  | 25.50 | 2.17 | Nauplius |  |
|  | 25.50 | 4.00 | Mysis |  |
|  | 25.50 | 5.08 | Protozoa |  |
|  | 28.00 | 0.59 | eggs | [65] |
|  | 29.00 | 1.97 | Nauplius |  |
|  | 29.00 | 2.96 | Mysis |  |
|  | 29.00 | 4.38 | Protozoa |  |
| *Penaeus setiferus* | 27.50 | 2.40 | Nauplius | [31] |
|  | 27.50 | 4.54 | Protozoa |  |
|  | 27.50 | 4.60 | Mysis |  |
| *Penaeus stylirostris* | 29.00 | 0.57 | eggs | [66] |
|  | 29.00 | 1.50 | Nauplius |  |
|  | 29.00 | 6.50 | Protozoa |  |
|  | 29.00 | 6.50 | Mysis |  |
|  | 28.25 | 0.63 | eggs | [67] |
|  | 28.25 | 2.08 | Nauplius |  |
|  | 28.25 | 3.71 | Mysis |  |
|  | 28.25 | 5.25 | Protozoa |  |
| *Penaeus vannamei* | 33.30 | 0.50 | eggs | [68] |
|  | 33.30 | 2.08 | Nauplius |  |
|  | 33.30 | 5.25 | Protozoa |  |
|  | 33.30 | 3.71 | Mysis |  |
|  | 29.50 | 0.53 | eggs | [69] |
|  | 29.50 | 1.60 | Nauplius |  |
| *Trachypenaeus curvirostris* | 26.00 | 0.63 | eggs | [70] |
|  | 26.00 | 1.71 | Nauplius |  |
|  | 26.00 | 3.07 | Mysis |  |
|  | 26.00 | 5.60 | Protozoa |  |
| *Pleoticus muelleri* | 20.00 | 1.00 | eggs | [71] |
|  | 20.00 | 1.98 | Nauplius |  |
|  | 20.00 | 7.00 | Mysis |  |
|  | 20.00 | 8.50 | Protozoa |  |

**Supplementary references of S2 File**

1. Boschi EE, Scelzo MA. Rearing the Penaeid shrimp *Artemesia longinaris* from egg to juvenile in the laboratory. *Proceedings of the annual meeting - World Mariculture Society*. 1974;5(1–4):443–444. doi: 10.1111/j.1749-7345.1974.tb00211.x

2. Cockcroft AC. The larval development of *Macropetasma africanum* (Balss, 1913) (Decapoda, Penaeoidea) reared in the laboratory. *Crustaceana*. 1985; 49(1): 52–74.

3. Choi Jh, Hong SH. Larval development of the kishi velvet shrimp, *Metapenaeopsis dalei* (Rathbun) (Decapoda: Penaeidae), reared in the laboratory*. Fishery Bulletin*. 2001; 99(2).

4. Chong VC, Sasekumar A. Larval development of the fiddler shrimp, Metapenaeopsis stridulans (Alcock, 1905) (Decapoda: Penaeidae) reared in the laboratory*. Journal of Natural History*. 1994; 28(6): 1265–1285. doi: 10.1080/00222939400770641

5. Hassan H. Early developmental stages of *Metapenaeus affinis* (Decapoda, Penaeidae) reared in a laboratory. *ICES Journal of Marine Science*. 1980; 39(1): 30–43.

6. Hudinaga M. On the nauplius stage of *Penaeopsis monoceros* and *Penaeopsis affinis*. *Jap J Zool.* 1941; 10(2): 305–393.

7. Muthu MS, Pillai NN, George KV. Larval development -*Metapenaeus affinis* (H. Milne Edwards). *CMFRI Bull.* 1979; 28: 40–49.

8. Thomas MM, Kathirvel M, Pillai NN. Spawning and rearing of the penaeid prawn, *Metapenaeus affinis* (H. Milne Edwards) in the laboratory*. Indian Journal of Fisheries*. 1974; 21(2): 543–556.

9. Tirmizi N, Hasan M-U, Kazmi QB. The larval development and spawning of *Metapenaeus affinis* (H. Milne Edwards) under laboratory conditions*. Pakistan Journal of Zoology*. 1981; 13: 141–155.

10. Preston NP. The combined effects of temperature and salinity on hatching success and the survival, growth, and development of the larval stages of *Metapenaeus bennettae* (Racek & Dall). *Journal of Experimental Marine Biology and Ecology*. 1985; 85(1): 57–74.

11. Rao GS. Larval development - *Metapenaeus brevicornis* (H. Milne Edwards*). CMFRI Bulletin.* 1979; 28: 60–64.

12. Teng SK. Observations on certain aspects of the biology of *Metapenaeus brevicornis* (H. Milne-Edwards) and *Penaeus merguiensis* (de Man) in the Brunei Estuarine system. Singapore: University of Singapore; 1971.

13. Crisp JA, Tweedley JR, D’souza FML, Partridge GJ, Moheimani NR. Larval development of the western school prawn *Metapenaeus dalli* Racek, 1957 (Crustacea: Decapoda: Penaeidae) reared in the laboratory*. Journal of Natural History*. 2016; 50(27–28): 1699–1724.

14. Muthu MS, Pillai NN, George KV. Larval development - *Metapenaeus dobsoni* (Miers). *CMFRI Bull.* 1979; 28: 30–39.

15. Thomas MM, Kathirvel M, Pillai NN. Observations of the spawning and rearing of *Metapenaeus dobsoni* under laboratory conditions*. Indian Journal of Fisheries*. 1974; 21(2): 575–579.

16. Leong PKK, Chu KH, Wong CK. Larval development of *Metapenaeus ensis* (de Haan) (Crustacea: Decapoda: Penaeidae) reared in the laboratory*. Journal of Natural History.* 1992; 26(6): 1283–1304.

17. Ronquillo JD, Saisho T. Early developmental stages of greasyback shrimp, *Metapenaeus ensis* (de Haan, 1844) (Crustacea, Decapoda, Penaeidae*). Journal of Plankton Research*. 1993; 15(10): 1177–1206.

18. Lee BD, T.Y L. Studies on the larval development of *Metapenaeus joyneri* (Miers*). Publication of the Marine Laboratory of the Pusan Fisheries College*. 1969; 2(19–25).

19. Preston NP. Some Factors Affecting the Survival of the Larvae of the Penaeid Prawns Penaeus Plebejus (Hess), *Metapenaeus macleayi* (Haswell) and *Metapenaeus bennettae* (Racek & Dall): University of Sydney; 1985.

20. Aktas M, Çavdar N. The combined effects of salinity and temperature on the egg hatching rate, incubation time, and survival until protozoal stages *of Metapenaeus monoceros* (Fabricius) (Decapoda: Penaeidae). *Turk J Zool*. 2012; 36(12): 249–253.

21. Courties C. Description des premiers stades larvaires de trois crevettes pénéides pêchées à Madagascar*: Penaeus indicus* H. Milne Edwards; *Penaeus semisulcatus* De Haan; *Metapenaeus monocero*s (Fabricius*). Cahiers Orstom Oceanographie*. 1976; 14: 49–70.

22. Mohamed KH, Vedavyasa Rao P, George MJ. Postlarvae of penaeid prawns of southwest coast of India with a key to their identification. FAO World scientific conference on the biology and culture of shrimps and prawns; Mexico 1967.

23. Khafage AR, Abdel Razek FA, Gobashy AFA, Taha SM. Early larval development of Egyptian metapenaeid shrimp, *Metapenaeus Monoceros* (Fabricius) reared under laboratory conditions*. Egypt J Aquat Res*. 2009; 35.

24. Kurata H, Vanitchkul P. Larvae and early postlarvae of a shrimp, *Metapenaeus burkenroadi*, reared in the laboratory. *Bulletin of the Nansei Fisheries Research Laboratory*. 1974; 7: 69–84.

25. Nandakumar G, Pillai NN, Telang KY, K. B. Larval development of *Metapenaeus moyebi* (Kishinouye) reared in the laboratory. *J Marine Biol Assoc India Cochin*. 1989; 31: 86–102.

26. Hassan H. Larval development of *Parapenaeopsis stylifera* decapoda penaeidae reared in a laboratory. *Journal du Conseil Conseil International pour l'Exploration de la Mer*. 1984; 41(3): 293–303.

27. Muthu MS, Pillai NN, George KV. Larval development - *Parapenaeopsis stylifera* (H. Milne Edwards*). CMFRI Bull.* 1979; 28: 65–74.

28. Thomas MM, George KV, Kathirvel M. On the spawning and early development of the marine prawn, *Parapenaeopsis stylifera* (H. Milne Edwards) in the laboratory*. Indian J. Fish*. 1975; 21(1): 266–271.

29. Heldt JH. La Reproduction chez les crustacés décapodes de la famille des Pénéides. *Annales de l’Institut. Océanographique de Paris,* 1938; 28: 1–206.

30. Kitani H. Larval development of naupliar stage of the northern brown shrimp *Penaeus aztecus* Ives and comparison with its earlier description. *Bull Japanese Soc Sci Fish*. 1986; 52: 1285–1288.

31. Krantz GE, Norris JP. Culture of pink shrimp, *Penaeus duorarum* Ives at the Turkey Point Experimental mariculture laboratory*. Sea Grant Tech Bull*. 1976; 36: 1–41.

32. Kitani H. Larval Development of the Crystal Shrimp *Penaeus* (*Farfantepenaeus*) *brevirostris* Under Laboratory Conditions. *Fisheries science*. 1997; 63(2): 218–227.

33. Kitani H, Alvarado N. The Larval Devlopment of the Pacific Brown Shrimp *Peneaus californiensis* HOLMES Reared in the Laboratory. *NIPPON SUISAN GAKKAISHI*. 1982; 48(3): 375–389.

34. Oka M. Studies on *Penaeus orientalis*, Kishinouye V. Fertilization and development*. Bull Fac Fish Nagasaki Univ*. 1967; 23: 71–87.

35. Dobkin S. Larvae of the pink shrimp (*Penaeus duorarum*). Presented at Proceedings of the Gulf and Caribbean Fisheries Institute 12, Miami (United States). 1960

36. Ewald JJ. The Laboratory Rearing of Pink Shrimp, *Penaeus duorarum* Burkenroad. *Bulletin of Marine Science*. 1965; 15(2): 436–449.

37. Kitani H. Larval Development of the Pink Shrimp *Penaeus duorarum* BURKENROAD Reared in the Laboratory and the Comparison with Earlier Descriptions. *NIPPON SUISAN GAKKAISHI*. 1985; 51(8): 1239–1248.

38. Fielder D, Greenwood J, Ryall J. Larval development of the tiger prawn, *Penaeus esculentus* Haswell, 1879 (Decapoda, Penaeidae), reared in the laboratory. *Marine and Freshwater Research*. 1975; 26(2): 155–175.

39. Muthu MS, Pillai NN, George KV. On the spawning and the rearing of *Penaeus indicus*  in the laboratory with a note on the eggs and larvae. *Indian Journal of Fisheries*. 1974; 21(2).

40. Hudinaga M. Reproduction, development and rearing of *Penaeus japonicus* Bate. *Jap J Zool*. 1942; 10: 305–393.

41. Hudinaga M, Miyamura M. Breeding of the 'Kuruma' prawn (*Penaeus japonicus* Bate). *J Oceanog Soc Japan*. 1962; 20(694–706).

42. Klaoudatos S. Breeding of *Penaeus kerathurus* larvae in the laboratory as a proposition to culture them on a commercial scale. *Thalassografika*. 1978; 2(1): 99–113.

43. Lumare F, Gozzo S. Osservazioni Sulla Morfogenesi Del Nauplio Di *Penaeus kerathuru*s Nelle Sue Relazioni Con Penaeus japonicus. Atti Soc Peloritana, *Scienze Fis Mat E Nat,* 165/175, 3/4, CNR-Roma1973.

44. Türkmen G. Larval Development of the Grooved Shrimp (*Penaeus kerathurus* Forskal, 1775) Under Laboratory Conditions. *Turkish Journal of Fisheries and Aquatic Sciences*. 2003; 3(2): 97–103.

45. Yúfera M, Rodriguez A, Lubián LM. Zooplankton ingestion and feeding behavior of *Penaeus kerathurus* larvae reared in the laboratory*. Aquaculture*. 1984; 42(3): 217–224.

46. Roberts SD, Dixon CD, Andreacchio L. Temperature dependent larval duration and survival of the western king prawn, *Penaeus* (*Melicertus*) *latisulcatus* Kishinouye, from Spencer Gulf, South Australia. *Journal of Experimental Marine Biology and Ecology*. 2012; 411: 14–22.

47. Shokita S. A note on the development of eggs and larvae of *Penaeus latisulcatus* Kishinouye, reared in an aquarium. *Biol Monogr Okinawa*. 1970; 6: 34–36.

48. Gopalakrishnan K. Larval rearing of red shrimp *Penaeus marginatus* (Crustacea*). Aquaculture*. 1976; 9: 145–154.

49. Motoh H, Buri P. Larvae of Decapod Crustacea of the Philippines-IV Larval Development of the Banana Prawn, *Penaeus merguiensis* Reared in the Laboratory*. NIPPON SUISAN GAKKAISHI*. 1979; 45(10): 1217–1235.

50. Zacharia S, Kakati VS. Optimal salinity and temperature for early developmental stages of *Penaeus merguiensis* De man. *Aquaculture.* 2004; 232(1–4): 373–382.

51. Motoh H. Larvae of Decapod Crustacea of the Philippines - III. Larval development of the giant tiger *prawn Penaeus monodon* reared in the laboratory. *Bull Japanese Soc Sci Fish*. 1979; 45(10): 1201–1216.

52. Reyes EP. Effect of temperature and salinity on the hatching of eggs and larval development of sugpo, *Penaeus monodon*. In: Taki Y, Primaevera JH, Llobrera JA , editors. Proceedings of the first international conference on the culture of penaeid prawns/shrimps; Iloilo City, Philippines; 1985.

53. Silas EG, Muthu MS, Pillai NN, George KV. Larval development - *Penaeus monodon* Fabricus. *CMFRI Bulletin*. 1979; 28: 2–11.

54. Villaluz DK, Villaluz A, Ladrera B, Sheik M, Gonzaga A. Reproduction, larval development and cultivation of sugpo (*Penaeus monodon* Fabricius*). Philipp J Sci.* 1969;98 (3–4): 205–233.

55. Kitani H. Larval Development of the Western White Shrimp *Penaeus occidentalis* Reared in the Laboratory. *Fisheries science.* 1996; 62(6): 883–891.

56. Boff MH, Marchiori MA. The effect of temperature on larval development of the pink shrimp *Penaeus paulensis*. Atlantica. 1984; 7: 7–13.

57. Lemos D, Phan NV. Ontogenetic variation in metabolism, biochemical composition and energy content during the early life stages of *Farfantepenaeus paulensis* (Crustacea: Decapoda: Penaeidae). *Marine Biology.* 2001; 138(5): 985–997.

58. Heng L, Rui-yu L. Comparative studies on the larval development of the penaeid shrimps, *Penaeus Chinensis, P. merguiensis* and *P. penicillatus. Chinese Journal of Oceanology and Limnology.* 1994; 12(4): 295–307.

59. Pan CH, Yu HP. Larval development of the red tailed prawn*, Penaeus penicillatus* reared in the laboratory*. Journal of the Fisheries Society of Taiwan*. 1990; 17(4): 247–265.

60. Pinto LG, Ewald JJ. Desarollo larval del camaron blanco, *Penaeus schmitti* Burkenroad, 1936*. Bol Cent Inves Biol Univ Sulia*. 1974; 12: 1–61.

61. Aktas M, Eroldogan OT, Kumluo M. Combined Effects Of Temperature And Salinity On Egg Hatching Rate And Incubation Time *Of Penaeus Semisulcatus* (Decapoda: Penaeidae). The Israeli *Journal of Aquaculture- Bamigeh*. 2004; 56(2): 124–128.

62. Devarajan K, Nayagam JS, Selvaraj V, Pillai NN. Larval development-IV*. Penaeus semisulcatus* de Haan. Larval development of Indian *Penaeus prawns*. *CMFRI Bull.* 1978; 28(4): 22–30.

63. Hassan H. The larval development of *Penaeus semisulcatus* de Haan, 1850 (Decapoda, Penaeidae) reared in the laboratory. *Journal of Plankton Research*. 1982; 4(1): 1–17.

64. Kungvankij P, Ruangpanit N, Dangsakul S, Chirastit C. An experiment on artificial propagation of *Penaeus semisulcatus* de Haan. Contribution No 2, Phuket Marine Fisheries Station. 1972; 23.

65. Ronquillo JD, Saisho T, McKinley RS. Early Developmental Stages of the Green Tiger Prawn, *Penaeus semisulcatus* de Haan (Crustacea, Decapoda, Penaeidae). *Hydrobiologi*a. 2006; 560(1): 175–196.

66. Kitani H. Larval Development of the Blue Shrimp *Penaeus stylirostris* STIMPSON Reared in the Laboratory*. NIPPON SUISAN GAKKAISHI*. 1986;52(7):1121–1130.

67. Prahl Hv, Gardeazábal M. Descripción de las larvas del camarón azul *Penaeus stylirostris* Stimpson. *Anales del Instituto de Investigaciones marinas de Punta Betín.* 1977; 9: 157–172.

68. Andrade-Vizcaino K. Descripcion del desarollo larval del camaron blanco *Litopenaeus stylirostris* (Boone, 1931), y evaluacion del indice de desarollo en funcion del regimen de alimentacion. La Paz: Universidad Autonoma de Baja California Sur; 2010

69. Kitani H. Larval Development of the White Shrimp *Penaeus vannamei* BOONE Reared in the Laboratory and the Statistical Observation of its Naupliar Stages*. NIPPON SUISAN GAKKAISHI*. 1986; 52(7): 1131–1139.

70. Ronquillo JD, Saisho T. Developmental Stages of *Trachypenaeus curvirostris* (Stimpson, 1860) (Decapoda, Penaeidae) Reared in the Laboratory*. Crustaceana.* 1995; 68(7): 833–863.

71. Iorio MI, Scelzo MA, Boschi EE. Desarrollo larval y postlarval del langostino *Pleoticus muelleri* Bate, mediante cultivos de laboratorio y muestras de plancton (Crustacea: Decapoda: Solonoceridae). *Sci Mar.* 1990; 45(4): 329–342.
