# Supplementary material for: Modeled buoyancy of eggs and larvae of the deep-sea shrimp Aristeus antennatus (Crustacea: Decapoda) in the northwestern Mediterranean Sea
Source: PLoS One. 2020 Jan 29;15(1):e0223396. doi: 10.1371/journal.pone.0223396 (PMC6988965; doi:10.1371/journal.pone.0223396)
Supplement: S1 Table — Percentage of buoyant stages reaching the surface or the Mixed Layer Depth (MLD) for a given density (kg/m3) in Individual-Based Model modified by the release period (early summer and late summer) and by the activation of random turbulences. (DOCX) [file pone.0223396.s006.docx]

**S1 Table. Frequence of surfacing individuals (in percentage) in preliminary experiments.**

| **Release period** | Early summer | | | | | | Late summer | |
| --- | --- | --- | --- | --- | --- | --- | --- | --- |
| **Buoyant stage** | eggs | | eggs | | eggs and nauplius | | eggs | |
| **Turbulence** | No | | Yes | | No | | No | |
|  | **Surface** | **MLD** | **Surface** | **MLD** | **Surface** | **MLD** | **Surface** | **MLD** |
| **800** | 4.39 | 4.12 | 4.78 | 4.24 | 0.00 | 0.00 | 4.78 | 4.24 |
| **810** | 3.10 | 2.92 | 3.38 | 2.94 | 0.00 | 0.00 | 3.38 | 2.94 |
| **820** | 4.03 | 3.67 | 4.06 | 3.74 | 0.00 | 0.00 | 4.06 | 3.74 |
| **830** | 4.54 | 4.39 | 4.82 | 4.56 | 0.00 | 0.00 | 4.82 | 4.56 |
| **840** | 5.55 | 5.21 | 5.90 | 5.32 | 0.00 | 0.00 | 5.90 | 5.32 |
| **850** | 6.86 | 6.53 | 7.33 | 6.71 | 0.00 | 0.00 | 7.33 | 6.71 |
| **860** | 8.38 | 7.86 | 8.66 | 8.05 | 0.01 | 0.02 | 8.66 | 8.05 |
| **870** | 9.48 | 9.14 | 9.82 | 9.40 | 0.03 | 0.03 | 9.82 | 9.40 |
| **880** | 10.66 | 10.39 | 10.80 | 10.60 | 0.02 | 0.04 | 10.80 | 10.60 |
| **890** | 10.97 | 10.88 | 11.06 | 11.17 | 0.05 | 0.09 | 11.06 | 11.17 |
| **900** | 10.36 | 10.91 | 9.91 | 10.65 | 0.11 | 0.20 | 9.91 | 10.65 |
| **910** | 9.02 | 9.62 | 8.37 | 9.25 | 0.24 | 0.54 | 8.37 | 9.25 |
| **920** | 6.70 | 7.30 | 6.03 | 7.01 | 0.61 | 1.14 | 6.03 | 7.01 |
| **930** | 3.70 | 4.28 | 3.28 | 3.95 | 1.32 | 2.85 | 3.28 | 3.95 |
| **940** | 1.66 | 1.98 | 1.38 | 1.78 | 3.34 | 7.25 | 1.38 | 1.78 |
| **950** | 0.51 | 0.68 | 0.36 | 0.56 | 8.07 | 16.26 | 0.36 | 0.56 |
| **960** | 0.09 | 0.12 | 0.07 | 0.09 | 17.64 | 30.08 | 0.07 | 0.09 |
| **970** | 0.00 | 0.00 | 0.00 | 0.00 | 30.83 | 31.76 | 0.00 | 0.00 |
| **980** | 0.00 | 0.00 | 0.00 | 0.00 | 29.93 | 9.50 | 0.00 | 0.00 |
| **990** | 0.00 | 0.00 | 0.00 | 0.00 | 7.66 | 0.22 | 0.00 | 0.00 |
| **1000** | 0.00 | 0.00 | 0.00 | 0.00 | 0.14 | 0.00 | 0.00 | 0.00 |

Percentage of buoyant stages reaching the surface or the Mixed Layer Depth (MLD) for a given density (kg/m^3^) in Individual-Based Model modified by the release period (early summer and late summer) and by the activation of random turbulences.
