# Supplementary material for: Modeled buoyancy of eggs and larvae of the deep-sea shrimp Aristeus antennatus (Crustacea: Decapoda) in the northwestern Mediterranean Sea
Source: PLoS One. 2020 Jan 29;15(1):e0223396. doi: 10.1371/journal.pone.0223396 (PMC6988965; doi:10.1371/journal.pone.0223396)
Supplement: S2 Table — Three eddies were shaped by the larval dispersal simulation (see Fig 5). Coordinates of the eddy center were estimated with an algorithm from [34]. The edge of eddies was got from the streamlines. Computation of the eddy radius is based on the circle surface (Area = pi*Radius2 and approximated the estimated eddy size. (DOCX) [file pone.0223396.s007.docx]

**S2 Table. Characteristics of eddies involved in the larval dispersals.** Three eddies were indicated and shaped by the larval dispersal simulation (see Fig. 5)

| **Regions** | **Month** | **Coordinates eddy center** | **Area (km^2^)** | **Radius (km)** | **Rotation** |
| --- | --- | --- | --- | --- | --- |
| Northern part of the Valencian Gulf | July | 1.96º E; 40.67º N | 2550 | 29 | Clockwise |
|  | September | 2.04º E; 40.61º N | 4231 | 37 | Clockwise |
| Southern part of the Valencian Gulf | July | 0.35º E; 39.46º N | 3353 | 33 | Clockwise |
|  | September | 0.32º E; 39.33º N | 4897 | 39 | Clockwise |
| Eivissa Channel | July | 0.72º E; 38.70º N | 2977 | 31 | Anticlockwise |

Coordinates of the eddy center were estimated with an algorithm from [34]. The edge of eddies was got from the streamlines. Computation of the eddy radius is based on the circle surface (Area= pi*Radius^2^ and approximated the estimated eddy size.
